# Downward-Growing Neural Networks

**DOI:** 10.3390/e25050733

**Published:** 2023-04-28

**Authors:** Vincenzo Laveglia, Edmondo Trentin

**Affiliations:** 1 DINFO, Università di Firenze, Via di S. Marta 3, 50139 Firenze, Italy; 2 DIISM, Università di Siena, Via Roma 56, 53100 Siena, Italy

**Keywords:** deep neural network, deep learning, adaptive architecture, growing neural network, target propagation

## Abstract

A major issue in the application of deep learning is the definition of a proper architecture for the learning machine at hand, in such a way that the model is neither excessively large (which results in overfitting the training data) nor too small (which limits the learning and modeling capabilities of the automatic learner). Facing this issue boosted the development of algorithms for automatically growing and pruning the architectures as part of the learning process. The paper introduces a novel approach to growing the architecture of deep neural networks, called downward-growing neural network (DGNN). The approach can be applied to arbitrary feed-forward deep neural networks. Groups of neurons that negatively affect the performance of the network are selected and grown with the aim of improving the learning and generalization capabilities of the resulting machine. The growing process is realized via replacement of these groups of neurons with sub-networks that are trained relying on ad hoc target propagation techniques. In so doing, the growth process takes place simultaneously in both the depth and width of the DGNN architecture. We assess empirically the effectiveness of the DGNN on several UCI datasets, where the DGNN significantly improves the average accuracy over a range of established deep neural network approaches and over two popular growing algorithms, namely, the AdaNet and the cascade correlation neural network.

## 1. Introduction

Selecting the best architecture for a given learning task has always been an open issue in the training of deep neural networks (DNNs). Trial-and-errors and heuristic solutions still represent the state of the art. Therefore, the selection process is frustrating and heavily time-consuming, and it generally ends up in sub-optimal architectures.

Formally, given a learning task T represented in terms of the corresponding supervised training set $D=\{{\left(x_{j},y_{j}\right)}_{j=1}^{N}\}$, the following steps are required in the development of a feed-forward DNN capable of tackling T: (1) set the number of input and output units equal to the corresponding dimensionalities of the input and target outputs in D, respectively; (2) fix the form of the activation functions in the output layer, such that their codomain matches the range of the target values in D; (3) most crucially, fix the internal structure of the DNN (which utterly affects the computational capabilities of the model). This includes fixing the number of hidden layers, the size of each such layer, and the form of the corresponding activation functions. If dealing with many hidden layers, quasi-linear activation functions are advisable in order to overcome numerical issues such as vanishing gradients [1]. Once the architecture has been fixed, a proper learning strategy shall be implemented. This strategy involves the selection of other hyperparameters, including the mini-batch size, a drop-out value [2], the batch-normalization [3], etc. All in all, model selection is no straightforward process, and it typically involves (implicitly or explicitly) applying a computationally expensive search strategy.

This paper investigates a novel framework for the automatic completion of the aforementioned steps (2) and (3), that is, the data-driven adaptation of the DNN architecture and, implicitly, of the corresponding neuron-specific activation functions. The proposed framework is herein referred to as the downward-growing neural network (DGNN). The learning process in the DGNN unfolds deep architectures by means of local, piecewise training sub-processes in an incremental way, with no need for the usual overall backpropagation of partial derivatives throughout the whole DNN. Inherently, this can be seen as an instance of the divide-and-conquer strategy. Section 4 makes it explicit that the DGNN realizes a model of exploratory causal analysis capable of causal discovery [4,5].

Before presenting the DGNN, we begin the treatment by studying a motivating example (Section 1.1) and surveying the literature related to the present research (Section 1.2). The details of the proposed algorithm are presented in Section 2 (“Materials and Methods”). The latter introduces the first two novel target-propagation methods (Section 2.1), namely, the residual driven target propagation (Section 2.1.1) and the gradient-based target propagation (Section 2.1.2), used as building blocks for the DGNN growing and learning procedure (Section 2.2). The outcome of experiments conducted on datasets drawn from the UCI repository is reported in Section 3 (“Results”), where the DGNN is compared favorably with established DNN paradigms and growing algorithms. Section 4 (“Conclusions”) draws the concluding remarks and pinpoints major directions for future research work.

### 1.1. Motivating Example

Let us consider a feed-forward neural network, for instance, a multilayer perceptron (MLP). To fix ideas, assume that the MLP has three layers, namely, $L_{0}$, $L_{1}$, and $L_{2}$, where $L_{0}$ (the input layer) has *d* input units (*d* being the dimensionality of the feature space), the hidden layer $L_{1}$ has arbitrary size, and $L_{2}$ (the output layer) of 1-dimensional (i.e., there is only one output unit). The function realized by the MLP is $y=f_{2}\left(f_{1}\left(x\right)\right)$ where $f_{i}=\sigma \left(W_{i}x+b_{i}\right)$ is the layer-specific function, that is, a mapping $R^{d_{i-1}}\to R^{d_{i}}$ where $d_{i}$ is the *i*-th layer dimensionality (i.e., the corresponding number of units), and σ:R→R is the usual element-wise activation function. The function $f_{0}$ is associated to the input layer and, as such, is not considered for all practical intents and purposes (in fact, input signals do not undergo any transformation before being propagated forward through the network).

Let us consider a classification problem with two classes, say, $\omega_{1}$ and $\omega_{2}$, and let $T=\{{\left(x_{j},{\hat{y}}_{j}\right)}_{j=1}^{N},\;x_{j}\in R^{2}$, ${\hat{y}}_{j}\in \left\{0,1\right\}\}$ be a supervised dataset where ${\hat{y}}_{j}=0$ if $x_{j}$ is in class $\omega_{1}$ and ${\hat{y}}_{j}=1$ else. We write $\hat{y}$ to represent the network output when the network is fed with the generic input x. Finally, to fix ideas, we assume that a logistic sigmoid σ(.) is associated to the output neurons of the network.

Once the training has been completed, we can consider the network weights as constants. The *k*-th neuron in $L_{1}$ realizes the logistic regression $o_{k}=\sigma \left(x_{1}w_{k1}+x_{2}w_{k2}+b_{k}\right)$ which, in turn, realizes the *k*-th component of the layer-specific multi-dimensional function $f_{1}\left(.\right)$. Depending on x, $o_{k}$ is valued along the tails of σ(.) or in its middle range. Whenever the module of the connection weights is large, $o_{k}$ is valued along the tails of the sigmoid for most of the inputs x; that is to say, $o_{k}$ turns out to be close to either 0 or 1.

**Figure 1:**
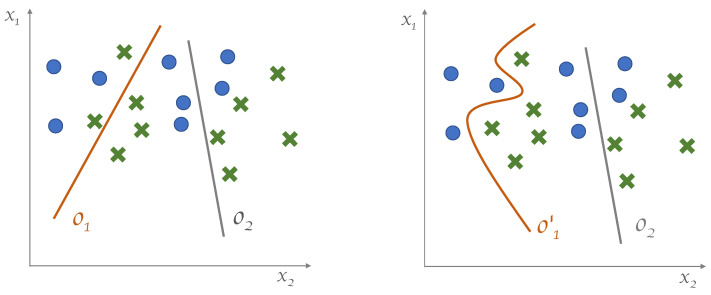
Data in $R^{2}$ belong to two classes, represented by crosses and circles. (**Left**): separation surfaces defined by two hidden neurons. (**Right**): separation surfaces expected to be generated by a growing model, where $o_{1}^{\prime }$ is the grown version of $o_{1}$.

We write $R_{0}$ to represent the decision region for class $\omega_{1}$, that is, the subspace of $R^{2}$ where $o_{k}$ is below the decision threshold (namely, $o_{k}\leq \frac{1}{2}$), and $R_{1}$ to represent the decision region for class $\omega_{2}$ (i.e., the subspace of $R^{2}$ where $whereo_{k}>\frac{1}{2}$). The set of points where $o_{k}=\frac{1}{2}$ forms the inter-class separation surface. Formally, we define the separation surface associated to the *k*-th neuron of the *i*-th layer as $S_{i}^{k}=\left\{x:F_{i}^{k}\left(x\right)=\gamma \right\}$, where $F_{i}\left(x\right)=f_{i}\left(f_{i-1}\ldots f_{0}\left(x\right)\right)$ and, in the specific case of sigmoid activation functions, $\gamma =\frac{1}{2}$. For sigmoids, a generic $x=(x_{1},x_{2})$ lies on the separation surface when $x_{1}w_{k1}+x_{2}w_{k2}+b_{k}=0$, that can be easily rewritten as

(1) $$x_{2}=-x_{1}\frac{w_{k2}}{w_{k1}}-\frac{b_{k}}{w_{k1}}$$

that is, the equation of a line having slope $-\frac{w_{k2}}{w_{k1}}$ and offset $-\frac{b_{k}}{w_{k1}}$. The computation realized by layer $L_{1}$ of the MLP is the set of the outputs of the neuron-specific logistic sigmoids for that layer. It is straightforward to see that this pinpoints which one of the two neuron-specific decision regions ($R_{0}$ or $R_{1}$) the generic input x belongs to, for each and every one of the individual neurons. A graphical representation is shown in Figure 1.

The argument we just used for $L_{1}$ can be extended to $L_{2}$, as well. The difference is that $L_{2}$ is fed with the outputs of $L_{1}$. Furthermore, $L_{2}$ being the output layer, its separation surface corresponds to the separation surface of the classifier. In general, we can say that the overall separation surface $S_{i}^{k}$ realized by the network is a function of the separation surfaces defined at the previous layer: $S_{i-1}^{1},S_{i-1}^{2},\ldots ,S_{i-1}^{d_{i-1}}$. In short, the output separation surface is $S_{2}^{k}=\phi \left(S_{1}^{1},\ldots ,S_{1}^{d_{1}}\right)$. In particular, in the simple architecture at hand, it is seen that $S_{2}^{k}$ is approximately in a piecewise linear form, where each linear segment corresponds to the separation surface generated in the preceding layer. It turns out that the overall separation surface is built from the linear segments realized by the neurons in $L_{1}$, as shown in Figure 1 (left).

In most of the cases, in practice, quasi-piecewise linear decision regions are not sufficient to separate data effectively. In this scenario, what we expect from a growing model is the capability to overcome such limitations by switching from piecewise-linear to generic decision surfaces. Furthermore, we want the model to define decision regions that can adapt to the model needs (i.e., to the nature of the data D and of the specific learning task T) in order to improve the performance of the resulting machine. This behavior is shown graphically in Figure 1 (right).

In case of input spaces having higher dimensionality, say, *m*, the outcome of the *k*-neuron is $o_{k}=\sigma \left(\sum_{h=1}^{m}w_{kh}x_{h}+b_{k}\right)$, and the equation realizing the separation surface is $\sum_{h=1}^{m}w_{kh}x_{h}+b_{k}=\gamma$, which is a hypercube of dimensionality m−1.

### 1.2. Related Works

The idea of learning/evolving the architecture of a neural network is not, per se, new. Early attempts date back to the late 1980s [6]. In this section, we review major, popular techniques for growing neural network architecture that relate somehow to the approach presented in this paper.

One of the most popular and effective paradigms for growing neural architectures is the cascade-correlation neural network [7]. It prescribes initializing the network as a minimal architecture with no hidden layers. After a first learning stage (based on gradient-descent), an iterative growing procedure is applied. It consists of adding a single neuron per time to the architecture. New forward connections are created, linking each pre-existing neuron (except for the output units) to the newly added neuron. The corresponding connection weights are learned by maximizing the correlation between the outcome of the new neuron and the output of the network. Afterwards, the values of these connection weights are clamped, and the output weights are learned via plain gradient-descent. Each new neuron partakes, in turn, in feeding the input to the next neuron to be added. This algorithm generates a particular instance of a deep architecture, where each hidden layer is composed of a single neuron and each internal neuron is fed from all the previous neurons (either input or hidden neurons).

An unsupervised incremental architecture-growing approach is represented by the growing neural gas [8], an extension of the traditional neural gas model [9]. A growing algorithm for semi-supervised learning is presented in [10]. As learning proceeds, more and more computational power is required of the learning machine to capture the input-to-output relationship encapsulated within the ever-increasing labeled fraction of the dataset. In [10], this is accomplished by creating new, additional layers that are plugged into the network architecture. Any such new layer is initialized as a replica of the previous one; then, a fine-tuning procedure is applied to optimize the parameters stemming from the introduction of the new layer.

A recent trend occurred in the development of algorithms capable of realizing dynamic architectures (including growing architectures) Recently, adaptive (e.g., growing) architectures proved suitable to continual learning setups (i.e., setups where the learning task changes over time). In particular, the approach presented in [11] exploits the knowledge encapsulated within a previously trained machine in order to train a new, larger neural architecture capable of modeling the new instances of the time-dependent learning task at hand.

A significant approach that relates to the present research is the AdaNet [12]. The AdaNet is initialized with a simple base-architecture, and new neurons are progressively added for as long as the performance improves. The criterion function to be minimized involves an architecture-driven regularized empirical risk where the complexity of the architecture plays the role of the regularization term. The growing stage goes as follows: given the base-architecture $h_{\ell }$ having *ℓ* layers, two candidate networks $h_{\ell }^{\prime }$ and $h_{\ell +1}^{\prime }$, are generated, having *ℓ* and ℓ+1 layers, respectively. The generic k+1-th layer in both candidate nets is fed with the output of the *k*-th layer in $h_{\ell }$, leveraging the embeddings of the data that $h_{\ell }$ learned already. Then, the candidate models undergo completion of their training process. Eventually, a new base-architecture is selected between $h_{\ell }^{\prime }$ and $h_{\ell +1}^{\prime }$ based on the corresponding performance in terms of the given loss function, and the whole procedure is iterated. It is seen that the computational cost of this growing algorithm can be very high, especially when the learning process ends up in large architectures.

Splitting steepest descent for growing neural architectures [13] is a technique that “splits” an individual neuron by replacing it with a set of new neurons whenever the learning process cannot improve the loss any further. Any such set of new neurons is initialized in such a way that the sum of their outputs equals the output of the neuron that underwent splitting. An ad hoc metric is defined for choosing the next neuron to be split. The approach was extended to the so-called firefly neural architecture descent in [14]. The latter sides the neuron-splitting mechanism with other growing tools that allow for the modification in width and depth of the neural architecture at hand.

Another approach called Gradmax was recently introduced in [15]. It focuses on an initialization procedure for the ingoing and outgoing connection weights of new neurons that have been introduced in the architecture at hand. The technique revolves around the idea of initializing the weights by solving an optimization problem such that (1) the output of the network is not initially affected by the activity of the new neurons, and (2) the gradient of the loss function with respect to the new weights is maximum in order to speed up the learning process.

## 2. Materials and Methods

**Figure 2:**
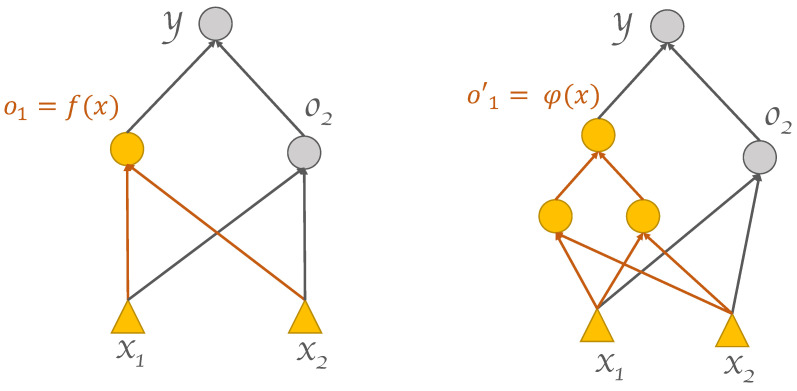
(**Left**): standard 1 hidden layer feed-forward network, also known as the base network. (**Right**): the grown network, after replacing the leftmost hidden neuron and its input connections with a subnet.

In light of the motivating example analyzed in Section 1.1, and relying on the notation introduced therein, we hereafter extend our scope to any generic supervised learning task. As we have seen, in a two-class classification task, the linear decision surface $S_{i}^{k}$ is the subset of the feature space where $o_{k}$ is equal to a certain value γ. In particular, in the case of sigmoid activation function, we have $\gamma =\frac{1}{2}$, and $o_{k}=\sigma \left(w_{k}^{in}x+b_{k}\right)$. The latter depends on the neuron input weights $w_{k}^{in}=\left(w_{k1},w_{k2}\right)$ and on the bias. We replace the neuron and all its input connections ($w_{k}^{in},b_{k}$) with a more general, adaptive processing component realizing a nonlinear activation function $\varphi :R^{d_{0}}\to R$ such that the corresponding decision surface $S^{k}$ results in a more flexible adaptive form. Such an adaptive processing component is realized via a smaller neural network Sub that we call subnet. Therefore, $o_{k}=\varphi \left(x;W\right)$ where W represents the set of all the weights of the subnet. In so doing, a modification of the original network architecture is achieved. Figure 2 shows a simple yet illustrative graphical example. Although the approach has been introduced referring to the illustrative setup outlined in Section 1.1 (two-class classification task over a two-dimensional feature space), it is seen that it can be applied in a straightforward manner, as is, to generic tasks having arbitrary input dimensionalities. The procedure can be repeated multiple times recursively, leading to a progressive growth of a DNN architecture with an arbitrary number of layers. Each application of the procedure replaces either (1) a group of the bottom-most hidden neurons in the original architecture with a subnet, or (2) a group of the bottom-most hidden neurons in a subnet with a sub-subnet, and so forth in a downward-growing manner. The following sections present the algorithmic building blocks used for realizing the DGNN growing and learning processes. These building blocks are in the form of techniques for propagating target outputs to hidden neurons within the DGNN, possibly located deep down in the network (Section 2.1), as well as in the form of procedures for generating and training the subnets involved (Section 2.2). Unfamiliar readers may find a gentle introduction to the basic notions of target propagation, architecture growing, and DNN refinement in [16].

### 2.1. Target Propagation

Let us first consider the regular backpropagation (BP) algorithm [17]. The core idea behind BP is that each weight of the network is partially accountable for the output error yielded by the model when fed with any labeled example $\left(x,\hat{y}\right)$ in the training set, where $\hat{y}$ is the target output over input x. Let $w_{i}$ be any generic connection weight in the DNN. Gradient-descent is applied in order to search for values of $w_{i}$ that reduce the output error. Therefore, $w_{i}$ is updated to its new value $w_{i}^{\prime }$ as follows:

(2) $$w_{i}^{\prime }=w_{i}-\eta \frac{\partial L}{\partial w_{i}}$$

where L is the loss function and η is the learning rate. In spite of its popularity and relevance, BP suffers from shortcomings when applied to deep architectures [1]. In particular, the backpropagated gradients tend to vanish in the lower layers of deep networks, hindering the overall learning process. A viable workaround was proposed in the form of Target Propagation (TP) [18,19], still an under-investigated research area. Originally proposed in [20,21] within the broader framework of learning the form of the activation functions, the idea underlying TP goes as follows. In plain BP, the signals to be backpropagated are related to the partial derivatives of the global loss function with respect to the layer-specific parameters of the DNN. To the contrary, in TP, the real target outputs (naturally defined at the output layer in regular supervised learning) are propagated downward through the DNN, from the topmost to the bottom-most layers of the network. In so doing, each layer gets explicit target output vectors that, in turn, define layer-specific loss functions that can be minimized locally without involving explicitly the partial derivatives of the overall loss function defined at the whole network level. As a consequence, the learning process is not affected by the numerical problems determined by repeatedly backpropagating partial derivatives throughout the DNN. In the TP scheme proposed hereafter, the targets are first computed for the topmost layer. Such output targets are then used for determining new targets for the DNN internal layers, according to novel downward-propagation techniques.

Given a DNN N having *ℓ* layers, let ${\hat{y}}_{\ell }$ represent the generic target output of the network, which is associated to the *ℓ*-th layer (the output layer). The aim of TP is the computation of a proper target value ${\hat{y}}_{\ell -1}$ for layer ℓ−1 and, in turn, for layers ℓ−2,ℓ−4,… In order to accomplish the task, a specific function ϕ(.) has to be realized, such that

(3) $${\hat{y}}_{\ell -1}=\phi \left({\hat{y}}_{\ell }\right)$$

When N is fed with an input vector x, the *i*-th layer of N (for i=1,…,ℓ, while i=0 represents the input layer which is not counted) is characterized by a state $h_{i}\in R^{d_{i}}$, where $d_{i}$ is the number of units in layer *i*, $h_{i}=\sigma \left(W_{i}h_{i-1}+b_{i}\right)$ and $h_{0}=x$ as usual. The quantity $W_{i}$ represents the weight matrix associated to layer *i*, $W_{i}\in R^{d_{i}\times d_{i-1}}$, $b_{i}\in R^{d_{i}}$ denotes the corresponding bias vector and $\sigma_{i}\left(.\right)$ represents the vector of the element-wise outcomes of the activation functions for the specific layer *i*. For notational convenience, the layer index will be omitted when it is not needed. Hereafter, we assume that the DNN at hand is based on activation functions that are in the usual form of logistic sigmoids. Nevertheless, the following results still hold for any kind of differentiable activation functions (upon minimal adjustments of the formalization). Let us consider a supervised training set $D=\{\left(x_{j},{\hat{y}}_{j}\right)|j=1,\ldots ,N\}$. Given a generic input pattern $x\in R^{n}$ and the corresponding target output $\hat{y}\in R^{m}$ both drawn from D, the state $h_{0}\in R^{n}$ of the input layer of N is defined as $h_{0}=x$, while the target state ${\hat{h}}_{\ell }\in R^{m}$ of the output layer is ${\hat{h}}_{\ell }=\hat{y}$. Relying on this notation, it is seen that the function $f_{i}\left(.\right)$ realized by the generic *i*-th layer of N can be written as

(4) $$f_{i}\left(h_{i-1}\right)=\sigma \left(W_{i}h_{i-1}+b_{i}\right)$$

Therefore, the mapping $F_{i}:R^{n}\to R^{d_{i}}$ realized by the *i*-th bottom-most layers over current input x can be expressed as the composition of the *i*-th layer-specific functions as follows:

(5) $$F_{i}\left(x\right)=f_{i}\left(f_{i-1}...\left(f_{1}\left(x\right)\right)\right)$$

Eventually, the function realized by N (which is an *ℓ*-layer network) is $F_{\ell }\left(x\right)$. Bearing in mind the definition of D, the goal of training N is having $F_{\ell }\left(x_{j}\right)≃{\hat{y}}_{j}$ for j=1,...,N. This is achieved by minimizing a point-wise loss function measured at the output layer. In the literature, this loss is usually the squared error, defined as $L\left(x_{j};\theta \right)={∥F_{\ell }\left(x_{j}\right)-{\hat{y}}_{j}∥}_{2}^{2}$, where θ represents the overall set of the parameters of N and ${∥\cdot ∥}_{2}$ is the Euclidean norm. Differently from the traditional supervised learning framework for DNNs, where the targets are defined only for the neurons of the output layer, TP consists in propagating the topmost layer targets $\hat{y}$ to the lower layers, in order to obtain explicit targets for the hidden units of the DNN as well. Eventually, gradient descent with no BP may be applied in order to learn the layer-specific parameters as a function of the corresponding targets. TP is at the core of growing and training the DGNN. Two TP algorithms are proposed in the next sections, namely, residual driven target propagation (RDTP) and gradient-based target propagation. The former applies to DNNs having a single output unit (e.g., binary classifiers), while the latter is suitable to arbitrary architectures.

#### 2.1.1. Residual Driven Target Propagation

Instead of attempting a direct estimation of the targets ${\hat{h}}_{\ell -1}$, hereafter we aim at estimating the *residual* values $z_{\ell -1}$ defined as the difference between the actual state $h_{\ell -1}$ and the desired, unknown target ${\hat{h}}_{\ell -1}$, such that $h_{\ell -1}+z_{\ell -1}={\hat{h}}_{\ell -1}$. The rationale behind using residuals is twofold:

1. Assume the network at hand, trained via plain BP over D, converges to the global minimum of the loss function. Under the circumstances, we would just let ${\hat{h}}_{i}=h_{i}$ such that $z_{\ell -1}={\hat{h}}_{\ell -1}-h_{\ell -1}$, for i=1,…,ℓ. To the contrary, residuals would not be null during the training: in particular, their module would start from a certain (large, in general) initial value and progressively converge to zero as training completes. Let *t* represent a certain training iteration, and let τ denote a certain number of consecutive training epochs. If the loss function decreases monotonically for t=1,2,…, it is immediately seen that $z_{\ell -1}\left(t+\tau \right)\leq z_{\ell -1}\left(t\right)$. Therefore, it is seen that after pre-training the network we have $|z_{\ell -1}\left|\ll \right|h_{\ell -1}|$, i.e., a smaller range of the inversion function ϕ(.), entailing a more error-robust target propagation technique.
2. In RDTP (as we will see shortly), whenever the network evaluated over a given input pattern results in a null error, then $z_{\ell -1}=0$ and ${\hat{h}}_{\ell -1}=h_{\ell -1}+0$; that is, the target reduces to the actual state. In so doing, the layer-wise training steps will not entail forgetting the knowledge learned by the DNN during the preceding pre-training process. This is not guaranteed by the established target propagation techniques.

Let us stick with the single-output network case for the time being. The core of RDTP lies in the estimation of the residues $z_{\ell -1}$ in the hidden layer ℓ−1, given the network output error ${\left(\hat{y}-y\right)}^{2}$. Once the residues are estimated, we define the target values for layer ℓ−1 as ${\hat{h}}_{\ell -1}=h_{\ell -1}+z_{\ell -1}$. Note that, for notational convenience, we omitted writing explicitly the dependence of the quantities on the input pattern. Relying on the notation introduced in Section 2.1, in the present scenario *ℓ* and ℓ−1 represent the output and the hidden layer of the DNN, respectively. Let us assume that a certain value of N is given. Using apexes and subscripts in order to point out the layer-specific and the neuron-specific indexes, respectively, the DNN output can be written as:

(6) $$y=\sigma_{\ell }\left(\sum_{u=1}^{d_{\ell -1}}w_{u}^{\left(\ell \right)}h_{u}^{\left(\ell -1\right)}+b^{\left(\ell \right)}\right)$$

with

(7) $$h_{u}^{\left(\ell -1\right)}=\sigma_{\ell -1}\left(\sum_{k=1}^{n}w_{u,k}^{\left(\ell -1\right)}x_{k}+b_{u}^{\left(\ell -1\right)}\right)$$

where $x\in R^{n}$ is the input pattern. A generic target output for the DNN is given by

(8) $$\hat{y}=\sigma_{\ell }\left(\sum_{u=1}^{d_{\ell -1}}w_{u}^{\left(\ell \right)}{\hat{h}}_{u}^{\left(\ell -1\right)}+b^{\left(\ell \right)}\right)$$

where ${\hat{h}}_{u}^{\left(\ell -1\right)}$ represents the target for *u*-th neuron in the hidden layer, for $u=1,\ldots ,d_{\ell -1}$. Since $z_{u}^{\left(\ell -1\right)}={\hat{h}}_{u}^{\left(\ell -1\right)}-h_{u}^{\left(\ell -1\right)}$, we can write ${\hat{h}}_{u}^{\left(\ell -1\right)}=h_{u}^{\left(\ell -1\right)}+z_{u}^{\left(\ell -1\right)}$ and the latter, in turn, can be rewritten as

(9) $$\begin{array}{cc} \hat{y} & =\sigma_{\ell }\left(\sum_{u=1}^{d_{\ell -1}}w_{u}^{\left(\ell \right)}\left(h_{u}^{\left(\ell -1\right)}+z_{u}^{\left(\ell -1\right)}\right)+b^{\left(\ell \right)}\right) \end{array}$$

(10) $$\begin{array}{cc} \; & =\sigma_{\ell }\left(\underset{\tilde{a}}{\underset{︸}{\sum_{u=1}^{d_{\ell -1}}w_{u}^{\left(\ell \right)}h_{u}^{\left(\ell -1\right)}}}+\underset{{\tilde{a}}_{z}}{\underset{︸}{\sum_{u=1}^{d_{\ell -1}}w_{u}^{\left(\ell \right)}z_{u}^{\left(\ell -1\right)}}}+b^{\left(\ell \right)}\right) \end{array}$$

where the quantities in the form ${\tilde{a}}_{z}$ are the activations (i.e., inputs) to the corresponding neurons, and will be defined shortly. Given the discussion so far, the target output can be written as

(11) $$\hat{y}=y+y_{z}$$

where $y_{z}$ is the output component related to the residues, namely, $y_{z}=\sigma_{\ell }\left({\tilde{a}}_{z}\right)$; therefore

(12) $$\begin{array}{cc} y_{z} & =\hat{y}-y \end{array}$$

(13) $$\begin{array}{cc} {\tilde{a}}_{z} & =\sigma_{\ell }^{-1}\left(y_{z}\right) \end{array}$$

Starting from ${\tilde{a}}_{z}$, that is, ${\tilde{a}}_{z}=\sum_{u=1}^{d_{\ell -1}}w_{u}^{\left(\ell \right)}z_{u}^{\left(\ell -1\right)}$, the residuals $z_{u}$ for the lower layer(s) of the DNN can be computed as follows. First, when a generic pattern x∈D is fed into the DNN, a certain output error $y_{z}$ is observed. Different neurons in layer ℓ−1 may have diverse degrees of responsibilities for that particular error. Formally, the responsibility $r_{u}^{\left(i\right)}\left(x\right)\in \left[0,1\right]$ of the *u*-th neuron in the generic *i*-th layer having size $d_{i}$ shall satisfy

(14) $$\sum_{u=1}^{d_{i}}r_{u}^{\left(i\right)}\left(x\right)=1$$

To this end, we define $r_{u}^{\left(i\right)}\left(x\right)$ as

(15) $$r_{u}^{\left(i\right)}\left(x\right)=\frac{\sigma_{i}\left(a_{u}\right)}{\sum_{k=1}^{d_{i}}\sigma_{i}\left(a_{k}\right)}$$

where $a_{u}$ and $a_{k}$ are the activations of the generic units *u* and *k*, respectively. In the following, for notational convenience, writing explicitly the dependence on x may be dropped whenever needed. Equation (15) allows for the computation of the residues $z_{u}^{\left(i\right)}$, $u=1,\ldots ,d_{i}$. It relies on the assumption that the higher the responsibility of a neuron on the overall error, the higher shall be the corresponding residue $z_{u}^{\left(i\right)}$ required to compensate for the misbehavior of the neuron at hand. Finally, we empirically factorize ${\tilde{a}}_{z}$ in terms of a sum of responsibility-weighted contributions from the neurons in the previous layer as ${\tilde{a}}_{z}=\sum_{u=1}^{d_{\ell -1}}w_{u}^{\left(\ell \right)}z_{u}^{\left(\ell -1\right)}$, where $r_{u}^{\left(\ell -1\right)}{\tilde{a}}_{z}=w_{u}^{\left(\ell \right)}z_{u}^{\left(\ell -1\right)}$, such that

(16) $$\begin{array}{c} z_{u}^{\left(\ell -1\right)}=\frac{r_{u}^{\left(\ell -1\right)}{\tilde{a}}_{z}}{w_{u}^{\left(\ell \right)}} \end{array}$$

The pseudo-code of the overall procedure is presented in Algorithm 1, where a pattern-specific index *j* is used in order to make explicit the dependence of each quantity on the specific input vector.
**Algorithm 1** Residual Driven Target Propagation (RDTP)**Input:** training set $D=\{{\left(x_{j},{\hat{y}}_{j}\right)}_{j=1}^{N}\}$, the network N, the output layer i+1**Output:** the propagated targets at layer *i*. For N, layer *i* corresponds to the single hidden layer.  1:**for** j=1 to *N* **do**  2:    $y_{j}\leftarrow F_{i+1}\left(x_{j}\right)$  3:    $y_{z,j}\leftarrow {\hat{y}}_{j}-y_{j}$  4:    ${\tilde{a}}_{z,j}\leftarrow \sigma_{i+1}^{-1}\left(y_{z,j}\right)$  5:    **for** u=1 to $d_{i}$ **do**  6:        $r_{u}^{\left(i\right)}\left(x_{j}\right)=\frac{\sigma_{i}\left(a_{u}\right)}{\sum_{s=1}^{d}\sigma_{i}\left(a_{s}\right)}$  7:        $z_{u}^{\left(i\right)}=\frac{r_{u}^{\left(i\right)}{\tilde{a}}_{z}}{w_{u}^{\left(i+1\right)}}$  8:        $h_{u}^{\left(i\right)}=\sigma_{i}\left(a_{u}\right)$  9:        ${\hat{h}}_{u}^{\left(i\right)}=h_{u}^{\left(i\right)}+z_{u}^{\left(i\right)}$10:    **end for**11:    ${\hat{h}}_{i,j}=\left({\hat{h}}_{1}^{\left(i\right)},...,{\hat{h}}_{d_{i}}^{\left(i\right)}\right)$12:**end for**

#### 2.1.2. Gradient-Based Target Propagation

By construction, RDTP can backpropagate targets to the (topmost) hidden layer only when the network has a single output unit. As a consequence, it is not suitable to further propagate targets from layer ℓ−1 to layer ℓ−2, unless layer ℓ−1 is one-dimensional (which is hardly the case). Therefore, RDTP can be applied only to traditional, single hidden layer MLPs having a single output unit. To overcome this limitation, an extended version of the algorithm is herein proposed, called gradient-based target propagation (GBTP). As in RDTP, the idea is to estimate the residual values $z_{\ell -1}$ for layer ℓ−1 such that ${\hat{h}}_{\ell -1}=h_{\ell -1}+r_{\ell -1}⊙z_{\ell -1}$, where the residues are multiplied element-wise by the responsibility of the individual neurons. In the following, the residues are computed via gradient descent by letting

(17) $${\hat{h}}_{\ell }^{\prime }=\sigma_{\ell }\left(W_{\ell }\left(h_{\ell -1}+r_{\ell -1}⊙z_{\ell -1}\right)+b_{\ell }\right)$$

and minimizing the loss $L\left({\hat{h}}_{\ell },{\hat{h}}_{\ell }^{\prime }\right)={∥{\hat{h}}_{\ell }-{\hat{h}}_{\ell }^{\prime }∥}_{2}^{2}$ by iteratively updating $z_{\ell -1}$ as

(18) $$z_{\ell -1}^{\prime }=z_{\ell -1}-\eta \frac{\partial L({\hat{h}}_{\ell },{\hat{h}}_{\ell }^{\prime })}{z_{\ell -1}}$$

that can be applied either in an online or batch fashion. Propagation of the targets to the preceding layers is straightforward, by iterating the procedure in a backward manner over the layer-specific parameters. Algorithm 2 presents the pseudo-code of GBTP, where the function calculate_layer_resp(net,i,x) computes the responsibilities for *i*-th layer of the network as in Equation (15), in an element-wise fashion, in response to the input x. The procedure $estimate\_residues(net,x,r_{i},{\hat{h}}_{i+1},h_{i})$ computes the residues for *i*-th layer by iterating the application of Equation (18) until a stopping criterion is met. Note that the pseudo-code uses a pattern-specific index for representing all the quantities involved in the computation.
**Algorithm 2** Gradient-based RDTP**Input:** training set $D=\{{\left(x_{j},{\hat{y}}_{j}\right)}_{j=1}^{N}\}$, the network N, the layer *i*
**Output:** the propagated targets at layer i−11:**for** j=1 to *N* **do**2:    **if** i=ℓ **then**3:        ${\hat{h}}_{i,j}\leftarrow {\hat{y}}_{j}$4:    **end if**5:    $h_{i-1,j}=F_{i-1}\left(x_{j}\right)$6:    $r_{i-1,j}$ = calculate_layer_resp($N,i-1,x_{j}$)7:    $z_{i-1,j}$ = estimate_residues($N,x_{j},r_{i-1},{\hat{h}}_{i,j},h_{i-1,j}$)8:    ${\hat{h}}_{i-1,j}\leftarrow h_{i-1,j}+r_{i-1,j}⊙z_{i-1,j}$9:**end for**

### 2.2. The Algorithm for Growing and Training the DGNN

Building on the TP mechanisms, it is straightforward to devise the DGNN growing and learning procedure. First, the DGNN is generated as a feed-forward neural network (e.g., an MLP) with a single hidden layer. Hereafter, we write *h-size* to denote the number of hidden units. Such an initial shallow neural network is trained via BP over the supervised data sample D. TP (either RDTP or GBTP) is then applied in order to estimate the target values for all the hidden neurons, and the corresponding values of the loss function is computed. Afterwards, the $\bar{k}$ hidden neurons having highest loss are selected (where $\bar{k}$ is a hyperparameter, see Section 3). These neurons and their input connections are replaced by a subnet Sub (this realizes the proper “growing step”). The subnet is built as a one-hidden-layer MLP having $\bar{k}$ output units (the *i*-th of which realizes the activation function $f_{i}\left(a_{i}\right)$ for the *i*-th neuron replaced by Sub), *h-size* hidden units, and as many input neurons as the dimensionality of the DGNN input layer. The subnet Sub is then trained via BP using the values yielded by TP as target outputs for the corresponding output neurons of Sub. The procedure is recursively applied: TP is used to estimate targets for the hidden neurons of Sub. For each neuron in the output layer of Sub, the corresponding loss is computed. The average loss values ${\bar{\lambda }}_{h}$ and ${\bar{\lambda }}_{Sub}$ are then determined by averaging over the losses yielded by the remaining original hidden units in the DGNN and by the output neurons of Sub, respectively. If ${\bar{\lambda }}_{Sub}\geq {\bar{\lambda }}_{h}$, then the $\bar{k}$ highest-loss neurons in the hidden layer of Sub are grown; otherwise, growing is applied to the $\bar{k}$ original hidden neurons having highest loss. In both cases, a new subnet is introduced (having the same architecture as Sub) and trained based on TP. The entire procedure is applied recursively to all the original hidden neurons that have not been grown yet, as well as to all the subnets already present in the DGNN. This recursive growing step is repeated until a stopping criterion is met (namely, an early sopping criterion based on the validation loss evaluated at the whole DGNN level). Finally, a global refinement [22] of the model may be carried out by means of an end-to-end BP-based retraining of the overall grown neural architecture over D (starting from the DGNN parameters learned during the growing process). The recursive growing procedure aims at developing architectures having a number of internal layers that suits the nature of the specific learning problem at hand. Instead of growing the architecture by simply adding new neurons to the single hidden layer, the DGNN expands individual neurons in a depth-wise manner. The highest-loss criterion for selecting the neurons to be replaced by subnets entails that only those portions of the architecture that are actually relevant to the learning task are eventually grown.

## 3. Results

Experiments were designed to (1) assess the effectiveness of the proposed approach, as well as to (2) demonstrate that the results achieved by the DGNN do compare favorably with (and, possibly improve over) the state-of-the-art techniques. Publicly available, popular datasets were used in the experiments. The datasets were drawn from the UCI repository [23]. They correspond to several application-specific classification problems, spanning a range of different underlying characteristics (namely, the dimensionality of the feature space, the nature of the features involved, and the cardinality of the dataset). The specific datasets used, along with their characteristics and their bibliographic source, are summarized in Table 1. All of them consist of real-life data collected in the field, corresponding to specific real-world tasks. The Adult dataset [24] is a collection of records from the Census database describing professionals in terms of age, work-class, education, race, sex, etc. The task is predicting whether the income of a given professional exceeds $50 K/annum or not. The Ozone dataset [25] consists of weather/climatic measurements (temperatures, wind speed, etc.) collected from 1998 to 2004 at the Houston, Galveston, and Brazoria area at 1–8 h intervals. The task is the detection of the ozone level. The Ionosphere dataset [26] is a collection of radar data collected by a phased array of 16 high-frequency antennas located in Goose Bay, Labrador. The task is the classification of the radar returns from the ionosphere as either positive (the radar returns show evidence of the presence of some type of structure in the ionosphere) or negative (returns do not show any such presence; the corresponding signals pass through the ionosphere). The Pima dataset [27] is a collection of diagnostic measurements (number of pregnancies the patient has had, BMI, insulin level, age, etc.) carried out at the US National Institute of Diabetes among female patients of Pima Indian heritage. The task is to diagnostically predict whether or not a patient has diabetes. The Wine dataset [28] is a sample of chemical analysis (such as the quantity of alcohol, of malic acid, of magnesium, etc.) over a number of wines grown in the same region in Italy but derived from different cultivars. The task is determining the origin of any given wine. Vertebral [29] is a biomedical dataset where orthopaedic patients are to be classified into three classes (normal, disk hernia, or spondylolisthesis) based on six biomechanical features. Finally, Blood [30] is a dataset extracted from the Blood Transfusion Service Center in the city of Hsin-Chu (Taiwan) that is used for the classification of a variety of different measurements characterizing different blood donations.

We adopted the same robust many-fold crossvalidation methodology (and the same partitioning of the UCI datasets into training, validation, and test sets, on a fold-by-fold basis) used in [31]. As in [31], the hyperparameters of each algorithm were selected using the validation fraction of each fold-specific subset. The hyperparameters were tuned via random search, selecting the specific configuration of hyperparameters that resulted in the minimum validation loss. Random search was applied to the selection of the DGNN hyperparameters as well. For all the training algorithms under consideration, the following stopping criterion was applied: stop training once the loss function evaluated on the fold-specific validation subset of the data has not shown a relative improvement of as much as (at least) 2% over the last 200 consecutive training epochs.

We first evaluated the improvement yielded by the DGNN over a standard DNN having the same initial architecture, namely, a three-layer DNN. Of course, the dimensionality of the input and output spaces are dataset-specific. The number of neurons per hidden layer was fixed according to the aforementioned model selection procedure applied to the plain DNN (the architecture was inherited by the DGNN as a starting point, before growing takes place), and it ranged between a minimum of eight neurons for the Adult dataset to a maximum of 30 neurons for the Ionosphere dataset. Table 2 compares the values of the average classification accuracies (and the corresponding standard deviations) yielded by the plain DNN with no growing mechanism (hereafter termed *base-model*) with the outcome of the *grown-model*, which is the same DNN whose architecture underwent growing during its training process. The accuracies are averaged over the test subsets of the many-fold crossvalidation procedure for the different UCI datasets under consideration. The third and fourth columns of the Table report the average absolute accuracy improvement offered by the grown-model over the base-model and the corresponding relative error rate reduction, respectively. It is seen that the DGNN yields an improvement over the base-model for all the UCI datasets at hand. The improvement is significant: in fact, it amounts to an average 25.66% relative error rate reduction. The average improvement of the DGNN over the plain DNN in terms of absolute accuracy is significant as well, being approximately 3%. For five out of seven datasets, the DGNN turned out to also be more stable than the base-model, resulting in a reduced standard deviation of the fold-by-fold dataset-specific accuracies. In fact, on average (last row of the Table), the standard deviation of the grown-model is smaller than the standard deviation of the base-model. This is remarkable in light of the fact that the DGNN ends up becoming a more complex machine than the bare base-model, and its increased complexity (and architectural variance) could have been suspected of worsening the generalization capabilities of the resulting learning machine, affecting the stability of the latter. These results are empirical evidence of the fact that this is not the case. To the contrary, it is seen that the DGNN training algorithm tends to grow subnets that actually improve the quality of the mapping realized by the DGNN without overfitting the specific training data.

Table 3 reports the average absolute accuracy improvement (%) and the average relative error rate reduction (%) yielded by the proposed growing mechanism as functions of the dataset-specific number of features. It is seen that the improvements offered by the DGNN are substantially independent of the dimensionality of the feature space. In fact, Pearson’s correlation coefficient *r* between the number of features and the average absolute accuracy improvement turns out to be r=−0.3395, which is a nonsignificant very small negative relationship between the two quantities (the *p*-value being equal to 0.4563). As for the correlation between number of features and average relative error rate cut, Pearson’s coefficient is r=−0.2317 (*p*-value =0.6172), i.e., an even less significant, very small negative correlation. In short, the performance of the DGNN growing algorithm is affected by the number of features to an extremely limited extent.

**Figure 3:**
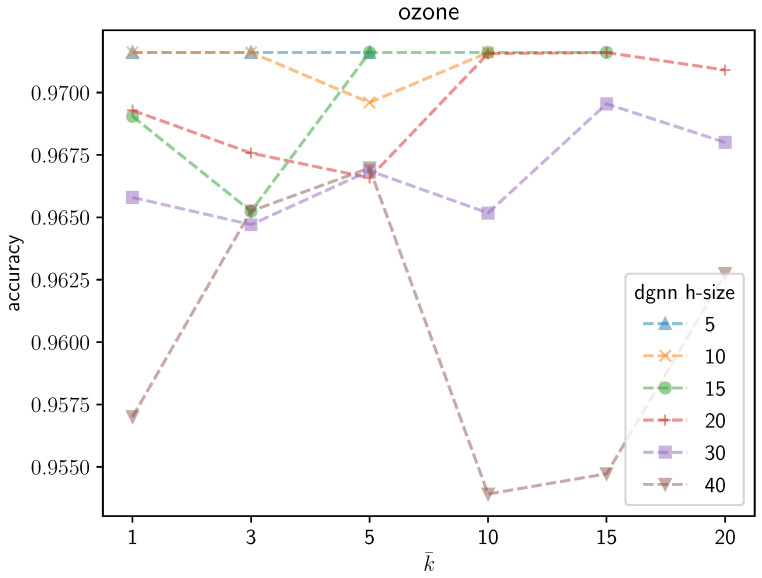
Accuracy yielded by the DGNN on the *Ozone* dataset as a function of the number $\bar{k}$ of neurons to be grown by the algorithm during the growing process, for different initial numbers of neurons (h-size) in the hidden layer.

**Figure 4:**
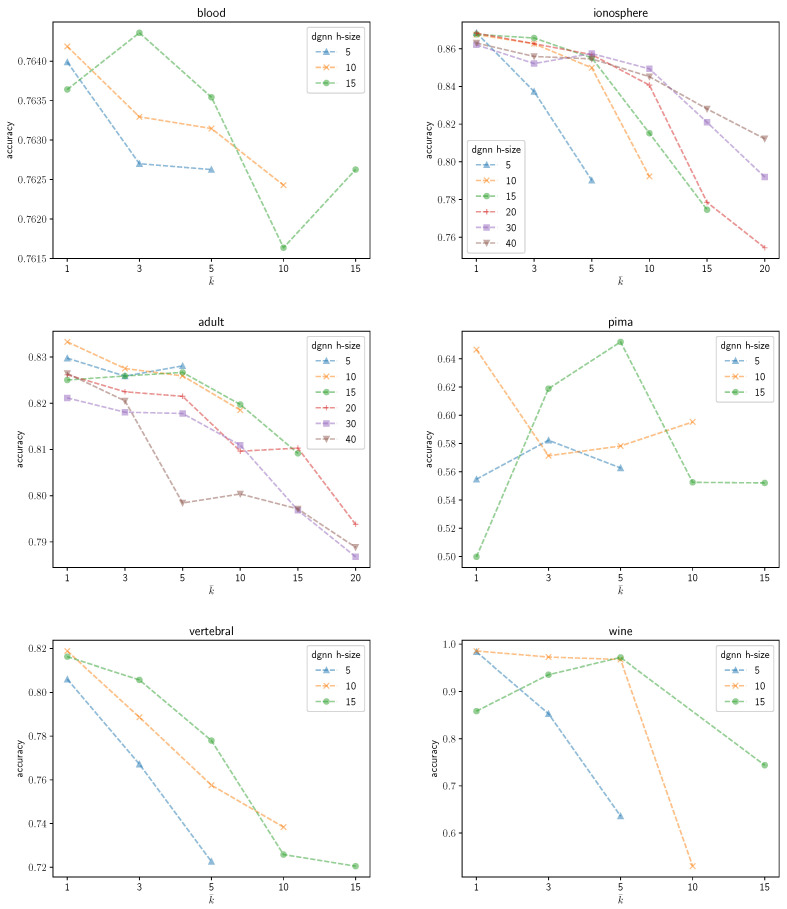
Accuracy of the DGNN on the UCI datasets as a function of $\bar{k}$.

An illustrative instance of the sensitivity of the DGNN performance to the number $\bar{k}$ of neurons that are grown during the learning process can be observed graphically in the following figures. Figure 3 shows the average validation accuracy on the *Ozone* dataset yielded by the trained DGNN as a function of $\bar{k}$. Different curves are plotted in the figure, each corresponding to a different initial number of neurons in the DGNN hidden layer. The following remarks are in order: (1) as expected, regardless of the growing mechanisms, the accuracy of the DGNN is affected significantly by the initial size of the hidden layer; (2) the optimal (i.e., yielding maximum-accuracy) value of $\bar{k}$ strictly depends on the initial dimensionality of the hidden layer; (3) the accuracy of the resulting model is definitely not a monotonic function of $\bar{k}$; neither does it present a unique maximum. Likewise, Figure 4 represents an overview of the variations of the validation accuracies on the remaining UCI datasets considered in the paper for different values of $\bar{k}$. A substantial variability in the behavior of the DGNN can be observed in the graphics, depending on the specific data at hand (and, implicitly, on the corresponding dimensionality), as expected.

The positioning of the learning and classification capabilities of the DGNN with respect to the state-of-the-art algorithms were assessed by means of two comparative experimental evaluations. The former aimed at putting the DGNN in the proper context of established results yielded by popular DNN-based approaches [32]. Hereafter, the established results are quoted from [32] (which obtained them via random-search model selection). Henceforth, the DGNN is compared with the following algorithm: self-normalizing neural networks (SNN) [32], sparse rectifier (s-ReLU) neural networks [33], deep residual neural networks (ResNet) [34], DNNs with batch-normalization (BN) [3], DNNs with weight normalization (WN) [35], and DNNs with layer normalization (LN) [36]. The outcomes of the experimental comparisons are reported in Table 4.

The results are reported in terms of average accuracy over the many-fold crossvalidation procedure, on a dataset-by-dataset basis. For each algorithm, the last row of the Table presents the algorithm performance (i.e., the average accuracy) averaged over the different datasets. For each dataset at hand, a boldface font is used in the Table to highlight the models that resulted in the highest average accuracy. It is seen that the DGNN resulted in the highest accuracy in five out of seven cases, and was second-best in the Ozone setup. In terms of overall average accuracy, the DGNN yielded a significant 1.56% gain over the ResNet, the latter being (on average) its closest competitor. Averaging over the six established DNNs reported in the Table, the DGNN resulted in an overall 2.66% average accuracy gain over its competitors. The two-tailed Welch’s *t*-test resulted in a confidence >75% of the statistical significance of the gap between the accuracies achieved by the DGNN and those yielded by the ResNet. The confidence increases (>88%) when comparing the DGNN with the remaining approaches.

Table 5 reports the comparison between the DGNN and the other DNNs in terms of average depth (number of layers) and average number of hidden neurons. Note that the input and output neurons are not counted, insofar as they are implicitly defined by the nature of the specific datasets under consideration; hence, they do not affect the comparisons. The quantities in the Table are averaged over the different UCI datasets and over the different many-fold crossvalidation iterations. Except for the DGNN case, the other values are quoted from [32]. A lower-bound on the average number of layers needed for the ResNet was estimated based on the latter being reported in [32] as a 6.35-block network on average, where each such a block involved a minimum of two layers. Since the number of layers and the number of hidden neurons are indexes of the complexity of the neural networks at hand, it is seen that the average DGNN complexity is dramatically smaller than for all the other DNNs. This results in improved generalization capabilities, which is likely to be at the core of the accuracy gains yielded by the DGNN according to Table 4. In short, the DGNN growing mechanism tends to grow only those neurons whose growth actually benefits the learning and generalization capabilities of the DNN undergoing growing.

In the second comparative experimental evaluation, we compared the DGNN with two established and popular growing algorithms for DNNs, namely, the adaptive structural learning of artificial neural networks (AdaNet) [12] and the cascade-correlation learning architecture (Casc-Corr) [7]. We adopted the same experimental setup used so far, namely, the same many-fold crossvalidation assessment procedure and the same model selection technique. The results are reported in Table 6. It turns out that the DGNN yields the highest average accuracies in four out of seven datasets, and the second-best accuracies in the remaining cases. When performing second-best, the difference between the accuracy yielded by the best scorer and the DGNN is negligible. In fact, upon averaging over the seven UCI datasets (last row of the Table) the DGNN yields the highest average accuracy overall. The two-tailed Welch’s *t*-test shows that the statistical significance of the improvement yielded by the DGNN over its closest competitor (that is, the Casc-Corr) is quite high, with a confidence >95%.

## 4. Conclusions

DGNNs extend plain DNNs insofar as they realize a growing mechanism that can adapt the architecture to the nature of the specific learning task at hand. Implicitly, such a growing mechanism results in the adaptation of the neuron-specific activation functions of the DNN, such that the activation function associated to a certain hidden neuron ξ is the (adaptive and nonlinear) function computed by the subnet associated with ξ. The best selling point of DGNNs over established growing algorithms lies in their locally expanding neurons only wherever necessary for improving the learning capabilities of the resulting machine, keeping the growth factor to a minimal scale which prevents the DNN from overfitting. From this perspective, DGNNs are complementary to the neural network pruning algorithms (which, by construction, start from an oversized architecture that is prone to overfitting since the early stages of the learning process).

The empirical evidence proves that the DGNN actually improves over established DNNs and growing algorithms, yielding sounder solutions to the different learning tasks covered in this paper. DGNNs offer practitioners a viable tool for compensating for possible mischoices at the architectural level made during the creation and initialization of the neural network. In principle, the DGNN growing mechanism may form the basis for overcoming any issues related to initial architectural choices.

An open issue with DGNNs is represented by the generation of target outputs for the different subnets. In fact, the target propagation process may not scale up to a very large dataset. Furthermore, the learning task entailed by the target propagation approach may also end up defining learning tasks for (at least some of) the subnets that are not necessarily simpler than the original learning task as a whole. Searching for solutions to these open problems is going to be part of the future work on the topic, alongside other research directions (e.g., the investigation of larger architectures for the subnets, the involvement of feature importance selection techniques for excerpting the neurons to be grown, etc.).

Finally, it is seen that the proposed approach introduces an implicit model of exploratory causal analysis, suitable to causal discovery [4,5] in the field. Let us resort to the probabilistic interpretation of artificial neural networks (the unfamiliar reader is referred to [37,38]). As shown in [39] (Section 6.1.3, page 202), a feed-forward neural network realizes an implicit model of the conditional probability p(t|x) of the targets t given the input x, where t and x are random vectors, insofar that y(x)=〈t|x〉=∫tp(t|x)dt. As observed in [40] (Section 5.6, page 272), the ANN may be used for solving either forward problems that “(*…*) correspond to causality in a physical system” (i.e., y(x) is caused by x), or inverse problems (e.g., in pattern recognition it is the class t that causes the probability distribution of the features x). Any generic hidden layer of the ANN realizes a nonlinear mapping φ(.) of the random vector x onto a latent random vector z=φ(x). The specific observation x causes z and the latter, in turn, causes y. Likewise, at each growing step the proposed algorithm introduces a new latent random vector that is caused by x and causes a selected subset of the components of the latent random vector yielded by the next hidden layer of the ANN, eventually causing y, in a cascade-like fashion. In short, the DGNN discovers spontaneously new latent variables and a chain of causalities that better explain the causality relationship between x and y. Moreover, since the algorithm selects subsets of the latent variables to be grown at each step, based on the extremization of the training criterion (that is, the best implicit fit of the resulting model to p(t|x)), eventually different causal chains (of different length, involving different latent and observable variables) are discovered. At a post-processing stage, since each latent variable discovered by the DGNN is realized via a corresponding sub-network defined over x, the connection weights in the sub-network that are below a given (small) threshold may be neglected, such that an overall pattern of causality between some input variables and specific latent and output variables emerges. Note that the forward-propagation of the input through such a grown DGNN complies with the two principles of Granger causality [41], insofar that (1) the feed-forward nature of the DGNN ensures that the causes happen prior to their effects, and (2) since the growing mechanism applies only to those variables that actually affect y and, in turn, the training criterion, the causes (along the causality chains) turn out to have unique information about the consequent values of their effects. It is seen that, due to its very architectural nature and dynamics, the DGNN and its generating data according to p(t|x) do relate to some extent to the notions of cellular automata and complex networks in the framework of causal calculus [42].

## Figures and Tables

**Table 1:** Characteristics of the datasets used in the experiments.

	Adult	Ozone	Ionosphere	Pima	Wine	Vertebral	Blood
Cardinality	48,842	2536	351	768	178	700	748
Nb. of features	14	72	34	8	13	6	5
Reference	[24]	[25]	[26]	[27]	[28]	[29]	[30]

**Table 2:** Average accuracy (±std. dev.) on the test subsets of the many-fold crossvalidation procedure yielded by the base-model and by the grown-model, respectively, along with the average absolute accuracy improvement offered by the latter over the former and the corresponding relative error rate cut.

Dataset	Base-Model	Grown-Model	Avg. Absolute	Avg. Relative
	Accuracy (%)	Accuracy (%)	Gain (%)	Error Cut (%)
Ionosphere	87.78 ± 2.03	93.47 ± 0.49	5.69	46.56
Wine	98.30 ± 0.98	99.43 ± 0.98	1.13	66.47
Vertebral	78.90 ± 2.96	87.01 ± 1.84	8.11	38.44
Blood	77.14 ± 0.58	80.35 ± 3.03	3.21	14.04
Pima	75.00 ± 2.68	77.08 ± 2.05	2.08	9.32
Ozone	97.24 ± 0.18	97.32 ± 0.27	0.08	2.90
Adult	85.38 ± 0.00	85.66 ± 0.00	0.28	1.92
*Average *	85.69 ± 1.34	88.62 ± 1.24	2.94	25.66

**Table 3:** Average absolute accuracy improvement and average relative error rate cut as functions of the number of features.

Dataset	Nb. of Features	Avg. Gain (%)	Avg. Error Cut (%)
Blood	5	3.21	14.04
Vertebral	6	8.11	38.44
Pima	8	2.08	9.32
Wine	13	1.13	66.47
Adult	14	0.28	1.92
Ionosphere	34	5.69	46.56
Ozone	72	0.08	2.90

**Table 4:** Comparison between the DGNN and the DNN-based classifiers: average accuracy on the different datasets. For each dataset, the highest accuracy is printed in bold.

Dataset	DGNN	SNN	s-ReLU	ResNet	BN	WN	LN
Ionosphere	93.47	88.64	90.91	**95.45**	94.32	93.18	94.32
Wine	**99.43**	97.73	93.18	97.73	97.73	97.73	97.73
Vertebral	**87.06**	83.12	87.01	83.12	83.12	66.23	84.42
Blood	**80.35**	77.01	77.54	80.21	76.47	75.94	71.12
Pima	**77.08**	75.52	76.56	71.35	71.88	69.79	69.79
Ozone	97.32	97.00	97.32	96.69	96.69	**97.48**	97.16
Adult	**85.66**	84.76	84.87	84.84	84.99	84.53	85.17
*Overall average*	**88.62**	86.25	86.77	87.06	86.46	83.55	85.67

**Table 5:** Comparison between the DGNN and the DNN-based classifiers: average depth (number of layers) and average number of hidden neurons.

	DGNN	SNN	s-ReLU	ResNet	BN	WN	LN
Depth	4.04	10.80	7.10	>12.70	6.00	3.80	7.00
Nb. of hidden neurons	35	2765	1818	1626	1536	973	1792

**Table 6:** Comparison between the DGNN and the established growing algorithms: average accuracy on the different datasets. For each dataset, the highest accuracy is printed in bold.

Dataset	DGNN	AdaNet	Casc-Corr
Ionosphere	**93.47 ± 0.49**	77.56 ± 4.05	91.75 ± 2.82
Wine	99.43 ± 0.98	94.90 ± 1.88	**100.00 ± 0.00**
Vertebral	**87.06 ± 1.84**	71.44 ± 5.44	**87.06 ± 1.84**
Blood	80.35 ± 3.03	63.62 ± **1.89**	**80.90** ± 3.62
Pima	**77.08** ± 2.05	62.88 ± 3.90	76.80 ± **1.54**
Ozone	97.32 ± 0.27	**97.40** ± 0.20	97.10 ± **0.17**
Adult	**85.66 ± 0.00**	82.30 ± 0.26	84.75 ± 0.25
*Average*	**88.62 ± 1.24**	78.59 ± 2.52	88.34 ± 1.46

## Data Availability

All data used in the paper are available publicly from the UCI Machine Learning Repository at https://archive.ics.uci.edu/ml/index.php (accessed on 31 January 2022).

## Abbreviations

The following abbreviations are used in this manuscript:

DNN	Deep neural network
AdaNet	Adaptive structural learning of artificial neural networks
BO	Bayesian optimization
BP	Backpropagation
BN	Deep neural network with batch-normalization
Casc-Corr	Cascade-correlation learning architecture
GBTP	Gradient-based target propagation
LN	Deep neural network with layer normalization
MLP	Multilayer perceptron
s-ReLU	Sparse rectifier linear unit
RDTP	Residual driven target propagation
ResNet	Deep residual neural networks
SNN	Self-normalizing neural networks
TP	Target propagation
WN	Deep neural network with weight normalization
